# Publics and their health: 50 years of continuity and change

**DOI:** 10.1093/pubmed/fdac086

**Published:** 2022-11-21

**Authors:** Alex Mold

**Affiliations:** London School of Hygiene and Tropical Medicine, Centre for History in Public Health, Keppel Street, London, WC1E 7HT, UK

**Keywords:** behaviour, chronic disease, communicable diseases

## Abstract

**Background:**

This article explores a set of changes and continuities in relation to public health and its publics in the UK since the establishment of the Faculty of Public Health in 1972.

**Methods:**

The article draws on historical research to produce a synthetic analysis of key changes and continuities in British public health since 1972.

**Results:**

Three key areas are identified. The first centres on the issue of who has responsibility for public health. The second examines the persistence of social and racial inequalities in population health. The third considers the ‘return’ of infectious disease as a threat to public health.

**Conclusions:**

Despite the trend to place more responsibility for individual and collective health on the public itself, there was a proliferation in the actors and authorities involved in securing and protecting the health of the public. The strong linkages between health and structural inequality, and the challenges of addressing these, demonstrate that public health never was (and never can be) solely an individual matter. The appearance of new diseases, such as HIV/AIDS and the return of ones thought to have been conquered, like tuberculosis, raised profound questions for public health authorities and the people they cared for.

In 1971, the year before the Faculty of Public Health was established, the leading cause of mortality in the UK was cardiovascular disease, accounting for half of all male deaths and 54% of female deaths.^1^ Almost 50 years later, in 2018, ischaemic heart disease was the leading killer for men, whereas Alzheimer’s and dementia were the leading cause of death for women.^2^ Setting aside changing terminologies as well as the difficulties of coding deaths and determining their causes, this pattern might suggest that population health in the UK had changed relatively little, at least in terms of mortality. But beneath this apparent continuity more profound shifts can be observed. Causes of death, of course, only tell a small part of the story. The public’s health, and ‘public health’ as an academic discipline, practice and set of services, have undergone significant changes over the last half century that reach beyond patterns of morbidity and mortality.

In this article I will explore a set of changes and continuities in relation to public health and its publics in the UK since 1972. I focus on three areas. The first concerns the question of who has responsibility for the public’s health. I suggest that despite the trend to place more responsibility for individual and collective health on the public itself, there was a proliferation in the actors and authorities involved in securing and protecting the health of the public. This is further underscored by my second area of focus: the persistence of social and racial inequalities in population health. The strong linkages between health and structural inequality, and the challenges of addressing these, demonstrate that public health never was (and never can be) solely an individual matter. The importance of the collective dimension to public health is not something likely to be lost on members of the Faculty of Public Health, but it did seem to come as surprise to some commentators during the Covid-19 pandemic. This brings me to my third area: the ‘return’ of infectious disease as a threat to public health. Although most deaths in the UK in the 1970s and in the 2020s (prior to the pandemic) were linked to non-communicable conditions, the appearance of new diseases, such as HIV/AIDS and the return of ones thought to have been conquered, like TB, raised profound questions for public health authorities and the people they cared for. Indeed, all three areas offer an opportunity to reflect not just on continuity and change in relation to the challenges facing public health, but also on the meaning of both the ‘public’ or ‘publics’ and ‘public health’ itself. This is a theme I (and others) have explored elsewhere, but it is worth returning to here as way to think about what has changed, what has not and why.^3^^,^^4^^,^^5^

## Responsibility for public health

The job of protecting the health of the public was never the responsibility of one single group of actors or authorities. Since at least the 19th century, government, public health officials, health professionals and the public themselves, all had a role to play.^6^ The extent to which individuals were thought to be to blame for public health problems was a consistent source of tension within public health policy and practice. In the 1930s, for instance, poverty underpinned many health conditions, but individual behaviour, such as poor parenting, was held up as a chief culprit by some authorities.^7^ From the 1970s onwards, however, individuals were held to be more responsible for their own health than they had been in the past. A prime example of this can be found in the UK Health Departments’ 1976 publication, *Prevention and Health Everybody’s Business.* The report asserted that ‘We as a society are becoming increasingly aware of how much depends on the attitude and actions of the individual about his [sic.] health. Prevention today is everybody’s business.’^8^ Indeed, the report concluded by stating that ‘Much of the responsibility for ensuring his [sic.] own good health lies with the individual.’^9^ Within the government, and academic public health, there was a growing consensus about the importance of chronic disease prevention, and especially the role of individual behaviour, as both cause and potential remedy for public health problems.^10^ This view was rooted in epidemiology and, perhaps to a lesser extent, ideology. From the 1950s onwards, numerous epidemiological studies demonstrated that there were links between individual behaviours, such as smoking, drinking alcohol and the consumption of fatty foods, to chronic health conditions such as cancer and heart disease. Persuading individuals to stop these activities, often through health education, was a way to improve individual and collective health. Such tactics also chimed with a broader political shift: the rise of neoliberalism and the ‘rolling back’ of the state from some areas of collective provision. A renewed focus on the entrepreneurial individual resulted in the ‘new public health’ or ‘healthism’.^11^^,^^12^ Here the public was recast as a set of rational, self-governing actors who could change their behaviours in line with advice and manage their own level of health risk accordingly.^13^^,^^14^

Although the impact of such changes can be overstated, few would doubt that individual behaviour has been a key focus for public health authorities over the past 50 years. The attention paid to individuals and their behaviours in relation to health, especially the consumption of certain products and substances, also highlighted the part played by other actors, such as commercial interests. For decades, the tobacco industry actively resisted any attempt to take responsibility for the burden of ill-health their products created, by suppressing and distorting evidence about the dangers of smoking.^15^^,^^16^ It has been suggested that other industries, including the alcohol industry and ‘big food’, have borrowed from the tobacco industry’s playbook to utilize similar tactics with their products.^17^^,^^18^^,^^19^ The damaging effect of these companies and their products on public health are clear, but the relationship between industry, government and the public is complex. The ability or desire of governments (whatever their political persuasion) to constrain public consumption is limited and operates within an environment where economic concerns are considered alongside the damage to public health. Nonetheless, the attention paid to the role of industry and other commercial determinants of health over the course of the past 50 years has broadened the range of actors thought to have some responsibility for the public’s health.^20^^,^^21^

On a practical level, who has direct responsibility for public health as a set of services, policies and practices have also expanded. As Samuel Trethewey notes elsewhere in this issue, ‘public health’ has occupied different positions within the UK health system and gone by different names. In 1972, when the Faculty of Public Health was founded, public health services and Medical Officers of Health as the individuals responsible for these were located within local government. Following the reorganization of the National Health Service (NHS) in 1973–4, public health was brought into the NHS, and re-named ‘community medicine’ with Medical Officers of Health becoming ‘community physicians’.^22^ Almost 40 years later, following the Health and Social Care Act of 2012, public health services returned to the ambit of local government in England.^23^ In Scotland, Wales and Northern Ireland public health remained within the NHS. The precise location of public health services within the health system was also further complicated by the fact that aspects of public health work were performed by other professionals and organizations. GPs and primary health care services were crucial to the treatment of individuals and the prevention of illness. Specific public health functions, like the design and delivery of health education, were devolved to specialist agencies such as the Health Education Council (1968–87), the Health Education Authority (1987–2000) and the Health Development Agency (2000–5). This proliferation of responsibility for improving public health, despite supposed individualization, suggests that collective elements of ‘public’ health were maintained over the last half century, albeit in different forms.

## Inequalities and health

A further counterweight to the view that individuals were solely responsible for their own health is offered by the persistence of social and racial inequalities in health over the past 50 years. Public health researchers had known that there was a relationship between socio-economic status and health since the mid-19th century, but in the latter part of the 20th century this became more prominent. By the late 1960s, it was clear that despite the optimism that had greeted the establishment of the NHS and other parts of the welfare state, health inequalities had not been eliminated. The so-called ‘rediscovery’ of poverty, and the sociologist Peter Townsend’s development of the notion of ‘relative poverty’ highlighted the fact that many people in the UK still struggled to attain a decent standard of living.^24^ The impact of socio-economic status on health was highlighted by a plethora of research including the famous Whitehall studies of the health of civil servants. A key paper from the first Whitehall study, published in 1978, showed that incidences of coronary heart disease (CHD) were linked to social class, with individuals in lower social classes more likely to have CHD than those in higher classes.^25^ This could not be explained by risk factors (such as smoking and diet) alone: the social structure itself had a negative impact on health.^26^

The importance of social structure in determining health outcomes was further underscored by the publication of the Black Report on inequalities in health in 1980.^27^ The report demonstrated that people in lower social classes had worse health, and that this applied at all stages of life from birth to death. Although the Black report was ignored by the Thatcher government, the existence of inequalities in health and what to do about them was not overlooked by public health researchers.^28^ In the 1990s, a second phase of the Whitehall study found a similar pattern of poor health linked to socio-economic status, something the researchers attributed to higher levels of stress amongst lower grade workers.^29^ Addressing social inequalities in health did come back onto the political agenda with the arrival of the Labour government in 1997, but as many commentators have pointed out, any improvements have long since stalled, and may even have worsened under austerity measures introduced by the coalition and Conservative governments in recent years.^30^^,^^31^ The Covid-19 pandemic also revealed considerable inequalities in health outcomes in the UK and elsewhere according to socio-economic status, as well as race and ethnicity.

The relationship between ‘race’ and health is complex and goes beyond a mirroring of social inequalities in health. Racialized understandings of health can be traced back to 19th century eugenics and tropical medicine, with certain ‘races’ deemed to be susceptible to certain conditions.^32^^,^^33^ The legacy of such views can be seen in more recent public health activities too. From the 1960s through the 1980s, there were a series of campaigns targeted at the British Asian community to reduce rates of rickets. Public health officials believed that children of Asian ethnic origin were more likely to have rickets as they did not eat fortified food, and they had darker skin that made the absorption of vitamin D through the skin from sunlight more difficult. Numerous health education campaigns highlighted the issue and attempted to persuade Asian mothers to change their children’s diet to include fortified foods and others rich in Vitamin D. But, as Roberta Bivins has pointed out, such campaigns were full of orientalist tropes and implied that rickets was due to the failure of British Asians to assimilate and adopt a ‘White’ diet.^34^^,^^35^ Although rickets was more common amongst British Asian children than amongst White British children, incidences were small. Moreover, there were much more common and serious health conditions within the Asian and non-Asian population to worry about. Indeed, other conditions, such as sickle cell anaemia, that were more prevalent amongst certain ethnic groups, were sometimes neglected and patients treated poorly.^36^ More recently, the Covid-19 pandemic demonstrated that even when conditions can affect all members of the population, not all people are affected equally, with a disproportionate impact on poor and BAME communities.

## The ‘return’ of infectious disease

The long-term legacy of Covid-19 for public health is still very much to be determined, but in the short-term it has brought wider attention to the problems posed by communicable disease. The epidemiologic transition from infectious to chronic disease in the early part of the 20th century, followed by the introduction of successful drug treatments in the mid-20th century, gave the appearance that infectious diseases had been conquered.^37^^,^^38^ By the latter part of the 20th century the arrival of new conditions challenged this view. In the past 30 years there were several epidemics of new, or newly recognized, infectious diseases. Of these, HIV/AIDS is the most significant. First identified in the early 1980s, HIV and AIDS were believed to pose a major threat to public health. Between 1981 and 2011, over 115 000 people were diagnosed with HIV in the UK, 27 000 of whom developed AIDS, and 20 000 people died.^39^ The development of combination anti-retroviral therapies in the late 1990s improved the prognosis of many people living with HIV and AIDS, and in high-income countries HIV/AIDS is now generally regarded as a chronic rather than acute disease. Even so, in 2019 it was estimated that 105 200 people were living with HIV in the UK, the majority of whom were receiving treatment for their condition.^40^ The story is very different for those living with HIV in low-income countries. Although the HIV/AIDS epidemic has, to some extent, been managed in high-income countries like the UK, it indicates the continuing susceptibility of all countries to new infectious diseases.

Indeed, there have been other outbreaks of previously unknown infectious diseases in recent years. In the 1990s, Bovine Spongiform Encephalopathy (BSE) or ‘mad cow disease’, prompted widespread concern. BSE, a degenerative brain disease found in cattle, was thought to have derived from a similar disease called Scrapie in sheep, raising the possibility that an infectious agent had crossed the species barrier. The concern was that this could happen again, this time with humans who ate infected meat. Such a fear appeared to be realized when new cases of a similar disease called Creutzfeldt–Jakob Disease, or CJD, were identified in younger people when previously it had only affected the elderly. Since 1996, 177 people in the UK have died from variant CJD, but due to its long incubation period this number could increase in future years.^41^ The presence of an infectious agent within the food chain also raises wider concerns about food safety and security.

Yet, of all the potential new infectious diseases, it had long been believed that a global flu pandemic was the most likely and possibly the most dangerous. There were several ‘near misses’ in recent years. The appearance of an especially virulent strain of avian flu, H5N1, in South-East Asia in 2003 caused alarm due to the large number of infected birds that died. H5N1 spread throughout Southeast Asia and into Europe. H5N1 crossed the species barrier and was contracted by humans in close contact with infected birds. Although the number of known global cases was small, 385, of these 218 people died, giving a case fatality rate of over 60%. In 2009–10, another flu strain, H1NI, or ‘swine flu’, did become a pandemic, but fortunately rarely led to serious illness, with 457 people dying in the UK by March 2010.^42^ Of course, it is not just flu that we need to be worried about. Novel coronaviruses started causing concern with the appearance of SARS in 2002, and then MERS, first identified in Saudi Arabia in 2012. The ability of another coronavirus, SARS-Cov-2, to cause a global pandemic has most certainly returned dealing with infectious diseases to the top of the public health agenda.

Yet, it is not just novel conditions that pose a problem. Some of the ‘old’ infectious diseases that we thought had been virtually eliminated are reappearing. In Britain, concern about a possible link between the Measles Mumps and Rubella (MMR) vaccine and childhood autism (now completely discredited) prompted thousands of parents to prevent their children from being immunised.^43^ Incidences of all these conditions increased, although they started to decline as confidence in the MMR vaccine returned.^44^ Other, supposedly beaten diseases also reappeared. Notifications of tuberculosis (TB) in England and Wales rose by 21% between 1988 and 1998.^45^ Some of these incidences of TB were linked to HIV/AIDS and other cases have been brought into the country by infected persons, but the increase is potentially significant. This is because several strains of TB are resistant to the antibiotics previously used to treat it. Other diseases, like some kinds of pneumonia, have also developed resistance to antibiotics. Conditions such as MRSA and C-Difficile (often acquired in hospital) are not only resistant to antibiotics but also appear to have developed partly as result of widespread antibiotic use. The WHO recently declared Antimicrobial Resistance (AMR) as one of the top 10 threats to global public health, and it is estimated that by 2050 as many as 10 million people a year could die because of AMR.^46^ In the future, it may no longer be possible to treat common infections with antibiotics, a prospect that has led the UN to suggest that the public health successes of the past may be undone.^47^^,^^48^

## Conclusion

The ‘back to the future’ world of a post-antibiotic era returns us to thinking about the long-running continuities and changes in public health over the past 50 years. The Faculty of Public Health was established at a time when public health, as a system, practice and academic discipline, was in flux. The relocation of public health functions within the English health service, the rise of lifestyle-related chronic disease and the professional consolidation conferred by the establishment of the Faculty were both a response to the conditions and an attempt to reimagine ‘public health’ and the ‘public’s health’ for a new era. Perhaps we are in a similar moment. The Covid-19 pandemic has shed new light on the three critical areas I pointed to, responsibility for public health; the persistence of inequalities; and the ‘return’ of infectious disease. Whatever lies ahead, it seems clear that working with publics is crucial to the success of public health.^49^ The Faculty of Public Health is in an unparalleled position to do just that.

## Notes

**Alex Mold**, Associate Professor
